# P-256. Development of Novel Clutter and Cleanliness Scales for Infection Prevention and Control in U.S. Home Healthcare Settings

**DOI:** 10.1093/ofid/ofae631.460

**Published:** 2025-01-29

**Authors:** Khadra Dualeh, Sasha Vergez, Nicole Onorato, Judith Brasch, Evette Ramos, Ashley Chastain, David Russell, Jinjiao Wang, Margaret V McDonald, Jingjing Shang

**Affiliations:** Columbia University School of Nursing, New York, New York; VNS Health, New York, New York; VNS Health, New York, New York; University of Rochester Medical Center, Rochester, New York; University of Rochester Medical Center, Rochester, New York; Columbia University School of Nursing, New York, New York; VNS Health, New York, New York; University of Rochester Medical Center, Rochester, New York; VNS Health, New York, New York; Columbia University School of Nursing, New York, New York

## Abstract

**Background:**

In the U.S., homebound older adults often have multiple chronic conditions thereby increasing their infection risk. For those receiving home health care (HHC), environmental hazards in the home can not only exacerbate infection risk but can also impede effective infection prevention and control (IPC) practices for HHC clinicians, patients and their caregivers. Timely and accurate assessment of environmental risks are crucial. Yet, no tools exist specifically for HHC clinicians to assess infection risk factors in the home environment. To fill this gap, we developed scales to measure clutter and cleanliness in HHC patients’ homes.

**Figure:**
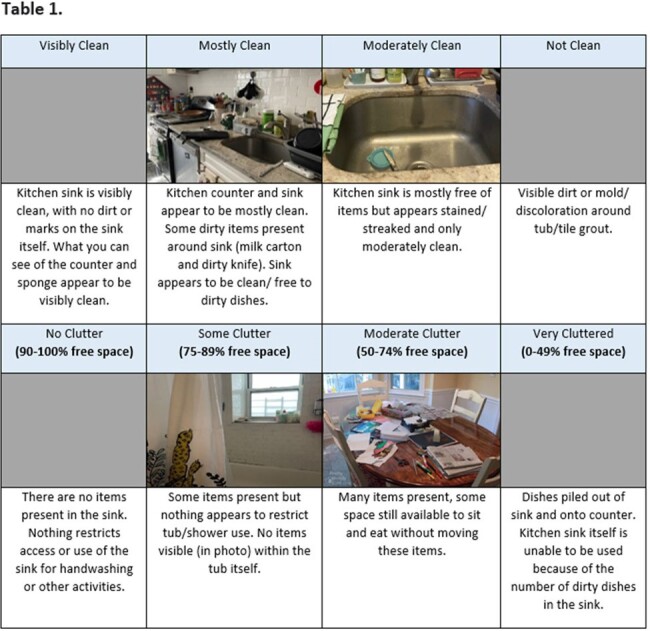

The clutter and cleanliness rating scale.

**Methods:**

First, we reviewed existing clutter and cleanliness scales, noting that most validated scales focused on hoarding. We adapted these scales and developed observational items focused on IPC in the home environment. During pilot testing with 15 HHC patients and caregivers from an urban HHC agency, we used a uniform rating scale for both clutter and cleanliness items, which posed challenges due to variable home environments and interviewer biases. Following pilot testing, we refined the scales based on interviewer feedback. To improve coding consistency, interviewers received training using a procedural manual and completed a home observation quiz with example pictures.

**Results:**

Our revised observational items now have distinct rating scales for cleanliness and clutter. Clutter is rated on percentages of free space to reduce subjectivity, and cleanliness is rated on a scale of visible clean to not clean (**Table 1**). Even with refinement, there was still difficulty achieving consistent ratings among interviewers, specifically with the middle scale points (mostly and moderately clean; some and moderate clutter). However, after scale revisions and interviewer trainings, home observation quiz responses among interviewers achieved at least 80% consistency.

**Conclusion:**

Our scales represent the first observational items designed to assess home cleanliness and clutter for IPC purposes. If widely adopted, these scales could enable HHC clinicians to effectively assess environmental hazards, informing patient/caregiver educational needs and enhancing patient safety by reducing infection risks.

**Disclosures:**

**All Authors**: No reported disclosures

